# Protective effects of hypercapnic acidosis on Ischemia–reperfusion-induced retinal injury

**DOI:** 10.1371/journal.pone.0211185

**Published:** 2019-01-25

**Authors:** Le-Tien Lin, Jiann-Torng Chen, Ming-Cheng Tai, Yi-Hao Chen, Ching-Long Chen, Shu-I Pao, Cherng Ru Hsu, Chang-Min Liang

**Affiliations:** 1 Department of Ophthalmology, Tri-Service General Hospital Songshan Branch, National Defense Medical Center, Taipei, Taiwan, Republic of China; 2 Graduate Institute of Medical Sciences, National Defense Medical Center, Taipei, Taiwan, Republic of China; 3 Department of Ophthalmology, Tri-Service General Hospital, National Defense Medical Center, Taipei, Taiwan, Republic of China; 4 Graduate Institute of Aerospace and Undersea Medicine, National Defense Medical Center, Taipei, Taiwan, Republic of China; University of PECS Medical School, HUNGARY

## Abstract

Ischemia–reperfusion (I/R) injury is associated with numerous retinal diseases, such as diabetic retinopathy, acute glaucoma, and other vascular retinopathies. Hypercapnic acidosis (HCA) has a protective effect on lung, myocardial, and central nervous system ischemic injury models. However, no study has evaluated its protective effects in an experimental retinal I/R injury model. In this study, retinal I/R injury was induced in Sprague Dawley rats by elevating the intraocular pressure to 110 mmHg for 60 minutes. HCA was induced before and after the injury. After 24 hours, the terminal dUTP nick end labeling assay was performed. Moreover, the ratios of cleaved caspase-3/total caspase-3, phosphorylated IκB/IκB, and phosphorylated p38 were measured through Western blotting. After 7 days, the rats’ aqueous humor was analyzed. In addition, electroretinography and retinal thickness measurement were performed in the rats. Moreover, the retinal neural cell line RGC-5 was exposed to 500 μM H_2_O_2_ for 24 hours to induce a sustained oxidative stress in vitro. The effects of HCA were evaluated by comparing oxidative stress, MAPK signals, NF-κB signals, survival rates, and apoptosis rates in the RGC-5 cells before and after H_2_O_2_ exposure. We further investigated whether the potent I/R-protective heat shock protein (HSP) 32 contribute to protective effects of HCA. Our results indicated that HCA has protective effects against retinal I/R injury both in vivo and in vitro, at multiple levels, including antiapoptotic, anti-inflammatory, antioxidative, and functional retinal cell protection. Further research clarifying the role of HCA in retinal I/R injury prevention and treatment is warranted.

## Introduction

Retinal ischemia–reperfusion (I/R) injury is a well-known pathological hallmark associated with diabetic retinopathy, ophthalmic artery occlusion, retinal vessel occlusion, and acute glaucoma, all of which result in severe visual impairment and subsequent vision loss [1]. Two phases occur after I/R injury, first within 24 hours after I/R injury and the second over the course of several days [2]. The major cause of the damage during the second phase (i.e., the reperfusion period) is the initiation of the inflammatory cascade, including free-radical-mediated retinal neuronal cell degeneration, cytokine release, and leukocyte activation [2–5].

Currently, reperfusion injury is thought to hinge on two critical events occurring during ischemia: energy failure caused by retinal blood flow disturbance and conversion of xanthine dehydrogenase to xanthine oxidase. During reperfusion, the sudden availability of oxygen to act as a cofactor enables xanthine oxidase to convert purine substrates accumulated during ischemia to uric acid, which generates superoxide anions and H_2_O_2_ as byproducts [5]. Consequently, retinal I/R injury contributes to a series of retinal cell insults, including overload of reactive oxygen species (ROS) and inflammatory cytokines [6, 7], increased vascular permeability [8], calcium influx, apoptosis of retinal neuron cells [9, 10], and impaired retrograde neurotrophin transport [11].

Determination of the mechanisms underlying retinal neural cell degeneration in I/R injury may facilitate the development of a treatment to protect the tissue against oxidative stress during reperfusion. ROS-induced oxidative stress is key in the pathophysiology of I/R injury [12, 13]. ROS also induce p38 mitogen-activated protein kinase (MAPK)-mediated tumor necrotic factor production and stimulate nuclear factor (NF)-κB signaling pathway to initiate a cytokine cascade through Toll-like receptors [14, 15].

Hypercapnic acidosis (HCA) is defined as the decrease in blood pH due to an excess of CO_2_ in the blood [16, 17]. HCA can be induced by adding CO_2_ (typically 5%–15%) into the inspired gas; it has been found to have a protective effect on lung, myocardial, intestinal, liver and central nervous system ischemic injury models [18–21]. In addition to organ inflammation, it may influence the apoptotic process [20]. Because CO_2_ is highly diffusible through tissues, HCA may modulate the apoptosis pathway systemically to various organs. We hypothesized that HCA reduces retinal I/R injury-induced inflammation and apoptosis.

The heat shock protein (HSP) family has long been associated with a generalized cellular stress response. Although HSPs typically appear to be protective, HSP32 [more commonly known as heme oxygenase (HO)-1] has emerged as potent protectants [22].

This study evaluated the HCA-induced anti-inflammatory effects, antioxidative effects, and apoptosis suppression in an experimental retinal I/R injury model and verified whether the potent I/R-protective HSP32 contribute to these protective effects of HCA.

## Materials and methods

### Induction of I/R injury in an animal model

All animal experiments were approved by and conducted under the guidance of the Institutional Animal Care and Use Committee (accredited by the Association for Assessment and Accreditation of Laboratory Animal Care International), National Defense Medical Center, Taipei, Taiwan (No: IACUC-15-366). All animal experiments were performed in compliance with the Association for Research in Vision and Ophthalmology Statement for the Use of Animals in Ophthalmic and Vision Research.

Eight-week-old male Sprague Dawley rats (LASCO, Charles River Technology, Taipei, Taiwan), weighing 250–300 g, were used in this study. These rats were housed in a temperature- and humidity-controlled animal room under a 12-hour light–dark cycle and supplied food and water ad libitum. All surgical procedures for inducing I/R injury were performed under aseptic conditions. The rats were placed on a temperature-controlled heated table to prevent hypothermia due to anesthesia. General anesthesia was induced through intraperitoneal injection of a mixture of 50 mg/kg ketamine (keTAlaR, Pfizer, Hsinchu, Taiwan) and 6 mg/kg xylazine (Rompun, Bayer, Gyeonggi-Do, Korea). After a rat became unconscious, we determined the depth of anesthesia by lightly pinching one foot pad to note for the presence of a reflex response. The surgical process was not performed until weak or no reflex was noted. Corneal analgesia was administered using a drop of topical 0.5% proparacaine hydrochloride ophthalmic solution (ALCAINE, Alcon, Puurs, Belgium). Pupils were dilated using 0.5% tropicamide + 0.5% phenylephrine ophthalmic solution (Mydrin-P, Santen, Osaka, Japan). Then, the anterior chamber of the left eye was cannulated with a 30-gauge needle connected to a saline reservoir at 150 cm above the eye, leading to a high IOP of 110 mmHg. Retinal ischemia and reperfusion were confirmed by fundus whitening and retinal blood flow restoration, respectively. The control group was treated with the same procedure, but with normal ocular tension. After removing the infusion needle from the anterior chamber for 60 minutes, the IOP returned to normal. An ophthalmic solution of norfloxacin (Nofoxin, MEDICINE, Taoyuan, Taiwan) and tobramycin (Tobrex, Alcon, Puurs, Belgium) was topically applied to the surgical eye before and after the procedure. Only the left eye of the rats was used in all experiments [1, 23].

### Animal group assignment and HCA setting

The rats were randomly divided into the following experimental groups: (1)control group, the surgical procedures for the induction of I/R injury were performed, but a saline reservoir connected to the anterior chamber cannula was kept at 30 cm above the eye; (2) HCA group, the rats received the same surgical procedures as the control group and then were exposed to HCA animal gas incubator for 1hour after surgery; (3) IR group, the rats were subjected to I/R injury; (4) HCA-IR group, the rats were exposed to HCA animal gas incubator for 1 hour before I/R injury induction; and (5) IR-HCA group, the rats were exposed to HCA animal gas incubator for 1 hour after I/R injury induction. Each group included 22 rats, of which 8 were used for the collection of aqueous humor (4 each were used for cell counting and the protein concentration assay), 4 were used for the terminal dUTP nick end labeling (TUNEL) assay, 6 were used for preparing retinal cell lysates, and the remaining 4 were used for ERG recording and histological analyses. To observe HCA effects, some rats used for ERG recording and histological analyses were sacrificed 7 days after I/R injury induction, whereas others were sacrificed 24 hours after HCA induction. The euthanasia of rats was conducted in CO_2_ chambers. After CO_2_ exposure, rats were placed in room air for 20 min to allow for possible recovery [24].

The gas composition of the HCA animal gas incubator settings was 5% CO_2_, 21% O_2_, and 74% N_2_. In HCA-induced rats, arterial blood gas analysis was performed in each rat under high partial CO_2_ pressure (PaCO_2_) such that pH and PaCO_2_ values were 7.2–7.4 and >60 mmHg, respectively.

### Cell count and protein concentration

Protein concentrations and cell counts in rats’ aqueous humor was measured, as described previously [25]. The aqueous humor was collected by puncturing the anterior chamber of the rats’ eyes with a 30-gauge needle, after which the rats were immediately euthanized. For cell counting, the aqueous humor was mixed with an equal amount of trypan blue solution (Sigma-Aldrich, St. Louis, MO, USA), and one drop of the cell suspension placed on a hemocytometer. The number of cells per square (equivalent to 0.1 μL) was counted manually under a light microscope; the mean number of cells counted from five squares per sample was multiplied by two to correct for the previous dilution. Total protein concentration in rats’ aqueous humor was measured using the bicinchoninic acid protein assay reagent kit (Pierce, Rockford, IL, USA).

### Terminal dUTP nick end labeling assay

TUNEL is a particularly useful technique for studying late-stage apoptosis [26]. To detect whether HCA reduces I/R injury-induced apoptosis, TUNEL assay was performed on retinal cryosections by using the TUNEL Apoptosis Detection Kit, according to the manufacturer instructions (Millipore, Bedford, MA, USA). The retinal cells were stained with 4′,6-diamidino-2-phenylindole (DAPI) to label nuclei. Images of TUNEL-positive cells were acquired through fluorescence microscopy (CKX41, Olympus Corporation, Tokyo, Japan). The number of TUNEL-positive cells was counted manually; the mean number of cells were counted from five different retina areas per sample.

### Electroretinography

Retinal physical functions were tested using electroretinography (ERG). The dark-adapted scotopic ERG was recorded using a recording device (UTAS-3000, LKC Technologies, Gaithersburg, MA, USA) 7 days after I/R injury induction. Before the monocular ERG, the rats were anesthetized through intraperitoneal injection of a mixture of 50 mg/kg ketamine and 6 mg/kg xylazine after 6 hours of dark adaptation. The pupils of the tested rats were dilated with 0.5% tropicamide + 0.5% phenylephrine ophthalmic solution, and a corneal anesthetic (topical 0.5% proparacaine hydrochloride ophthalmic solution) was applied. The rats were placed on a heating plate to prevent hypothermia due to anesthesia. Before a stainless-steel wire loop electrode was placed in contact with the cornea, the rats’ eyes were coated with 0.2% Carbomer ophthalmic gel (Vidisic, Dr. Gerhard Mann Chem.-Pharm. Fabrik, Berlin, Germany) to maintain corneal hydration. All procedures were prepared under dim red light for ERG recordings. Stainless needle electrodes were placed subcutaneously on the temporal canthus and the rats’ tail served as reference and ground electrodes, respectively. Light stimulations were produced using a full-field stimulation globe with an LED light source positioned 15 cm away from the eye. When the rats’ respiration became steady, the standard combined ERG (maximal combined response, cone and rod) were measured. Standard flash intensity was 3.0 cd-s/m^2^ with a duration of 2 ms, acquisition time was 200 ms, and the bandpass of amplifiers was 0.3–500 Hz for each registration. The amplitude of the α wave was measured from the prestimulus baseline to the trough of the ERG, whereas that of the β wave was measured from the trough to the peak. The difference of cursor of α and β waves was compared between groups.

### Histology

The eyes were enucleated from the rats which were euthanized on the 7th day after the induction of I/R injury for histology and immunohistochemistry assays. The eyes were immersion-fixed in 10% buffered formalin for at least 24 hours, after which they were embedded in optimal cutting temperature compound in cryomolds and snap-frozen in liquid nitrogen. Subsequently, serial axial cryostat sections (6 μm thick) were cut using the microtome portion of the cryostat from each eye, starting at the optic nerve head. The sections were placed on a glass slide and stained with hematoxylin and eosin.

### Cell culture

The retinal neural cell line RGC-5 [27], obtained from the American Type Culture Collection (Manassas, VA, USA), was grown in 1 mg/mL glucose Dulbecco’s modified Eagle’s medium (DMEM, Invitrogen-Gibco, Grand Island, NY, USA) supplemented with 4 mM L-glutamine, 10% fetal bovine serum (Invitrogen-Gibco), 100 U/mL penicillin, and 100 μg/mL streptomycin (Sigma-Aldrich). The cells were cultured in a humidified 37°C/5% CO_2_ incubator. The culture medium was replaced twice weekly.

### Induction of hypercapnia and oxidative stress

RGC-5 cells were seeded at a density of 10,000 cells/well in 6-well plates and incubated in 5% CO_2_ at 37°C for 24 hours. Normocapnia comprised standard incubator atmosphere: humidified 5%CO_2_ (pCO_2_ 36 mmHg)/95% room air, at 37°C. Hypercapnia consisted of 10% CO_2_ (pCO_2_ 72 mmHg)/21%O_2_/69%N_2_. The pH value of the culture medium was maintained between 7.25 and 7.38 [28]. Cells were exposed to hypercapnia for 1 hour before or after induction of oxidative stress. Cells were exposed to 500 μM H_2_O_2_ in serum-free DMEM for 24 hours to induce a sustained oxidative stress in vitro. Untreated cells and cells treated with H_2_O_2_ alone were used as normal and H_2_O_2_ control, respectively.

### Apoptosis assay through fluorescence-activated cell sorting

Flow cytometric identification was used in RGC-5 apoptosis analysis. The isolated cells were stained with fluorescein isothiocyanate-conjugated Annexin V and propidium iodide (PI) by using the Apoptosis Detection Kit (BD Biosciences Pharmingen, San Jose, CA, USA). Then, the viable cells were quantified to examine the effects of HCA induction before or after H_2_O_2_-induced oxidative stress. According to the manufacturer instructions, duplicates in each sample were run. Then confluent cultured cells were washed twice in phosphate buffer saline (PBS) and analyzed through flow cytometry on a fluorescence-activated cell sorting (FACS) flow cytometer (Becton, Dickinson, & Co., Sunnyvale, CA, USA).

### Preparation of rat retinal cells lysates

In each group, the rats’ retinas were rinsed with ice-cold PBS and carefully isolated by scraping. The detached tissues were homogenized in 50 μL of hypotonic buffer (10 mM HEPES-KCl, 1 mM β-mercaptoethanol, and 1 mM dithiothreitol) and then incubated on ice for 10 minutes. Subsequently, the homogenate was vortexed for 10 seconds and then centrifuged at 1000 rpm and the supernatant was discarded. The pellet was resuspended in 100 μL of lysis buffer [50 mM Tris-HCl (pH 7.5), 2% sodium dodecyl sulfate (SDS), 1 mM phenylmethylsulfonyl fluoride, and 10 μL/mL protease inhibitors], and incubated on ice for 10 minutes. The insoluble debris of rat retina lysates were removed by centrifugation at 12,000g for 15 minutes at 4°C.

### Preparation of RGC-5 cell lysates

First, the cell culture dish was placed on ice, and the cells were washed with ice-cold PBS. The PBS was aspirated; then, ice-cold lysis buffer [50 mM Tris-HCl (pH 7.5), 2% SDS, and 1 mM phenylmethyl sulfonyl fluoride] was added. Adherent cells were scraped off the dish using a cold plastic cell scraper, and then, the cell suspension was gently transferred into a precooled microfuge tube. Constant agitation was maintained for 30 minutes at 4°C. The suspension was centrifuged at 12,000g in a microcentrifuge at 4°C for 15 minutes. The tubes were gently removed from the centrifuge and placed on ice. The supernatant was aspirated and placed in a fresh tube kept on ice, and the pellet was discarded.

### Western blot

The total protein concentration of rat retina lysates,RGC-5 cell lysates, and nuclear extracts was measured using the bicinchoninic acid protein assay (Pierce), with bovine serum albumin as the standard. Equal amounts of the homogenates (20 μg of lysate) were resolved with one-dimensional 10% SDS polyacrylamide gel, separated electrophoretically, and transferred to a polyvinylidene difluoride membrane (Immobilon, Millipore). The membranes were blocked with 5% (wt/vol) milk in Tris-buffered saline (50 mM Tris-HCl, pH 7.4, and 150 mM NaCl) containing 0.05% Tween-20 for 1 hour at room temperature on a shaking table. The blots were incubated overnight at 4°C with primary antibodies directed against p38, which was purchased from BD PharMingen (San Diego, CA, USA). Antibodies against phosphorylated p38, caspase3, and cleaved caspase3 were obtained from Cell Signaling Technology (Beverly, MA, USA). Phosphorylated IκB-α and IκB-α antibodies were purchased from Santa Cruz Biotechnology (Santa Cruz, CA, USA); the membranes were washed and blotted with horseradish peroxidase-conjugated secondary antibody (1:1000; Jackson ImmunoResearch Laboratories, West Grove, PA, USA) for 1 hour at room temperature. The proteins were visualized on X-ray films using the standard enhanced chemiluminescence procedure, and the mean protein levels were determined densitometrically with ImageJ (version 1.46a; provided in the public domain by the National Institutes of Health, Bethesda, MD, USA).

### Measurement of HO-1 activity

The activity of HO in bulbal tissue was measured by the rate of appearance of bilirubin [29]. In brief, cellular extracts were added to the reaction mixture containing 2 mM glucose-6-phosphate, 0.2 U/ml glucose-6-phosphate dehydrogenase, 0.8 mM NADPH, 20 μM hemin, 2 mg of rat liver cytosol (source of biliverdin reductase), 2 mM MgCl_2_, 50 mM Tris-HCl, and pH 7.4. The mixtures (total volume: 1 ml) were incubated at 37°C for 60 minutes in the dark and then placed on ice to terminate the reaction. Bilirubin was extracted with chloroform, and bilirubin production was calculated as the difference between the absorbance at 464 nm and 530 nm (extinction coefficient for bilirubin: 40 mM^−1^ cm^−1^). HO activity was reported as picomoles of bilirubin formed per milligram of protein per hour. Differences of two activity measurements in two different samples with or without rat liver cytosol but from the same tissue type may be attributed to HO-1 activity because HO-2 is noninducible and constitutive.

### Statistical analysis

Descriptive statistics is expressed as mean ± standard error of the mean (SEM). The differences between the groups were analyzed by the χ^2^ test or Student’s *t* test, as appropriate. One-way analysis of variance was used for comparisons involving three or more group means. All statistical assessments were two-sided and evaluated at a significance level of 0.05. Statistical analyses were performed with the SPSS (version 18.0; SPSS, Chicago, IL, USA).

## Results

### HCA reduced I/R-induced cellular infiltration and protein concentration in aqueous humor

**Fig 1:**
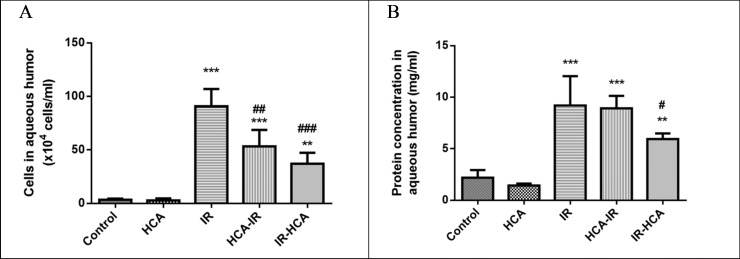
Numbers of cells (A) and protein concentrations (B) in the aqueous humor. The results are the mean ± SEM of three independent experiments. ***P < 0.001 versus control group, **P < 0.01 versus control group; ###P < 0.001, ##P < 0.01, #P < 0.05 versus IR group.

Inflammation in the anterior chamber of the eye increases the number of cells and protein concentration of the aqueous humor [30]. To examine HCA efficacy in attenuating inflammatory reaction in the rats subjected to I/R injury, we investigated the cell number and protein concentration changes in the anterior segment of the eye. In the IR group, obvious inflammatory reaction was observed; moreover, anterior chamber transparency improved in the HCA-IR and IR-HCA groups. Consistent with the expectation, the HCA induction attenuated the I/R injury-induced inflammation in the anterior segment of the eye. As shown in Fig 1A, the number of infiltrated cells in the aqueous humor of the IR group was significantly increased [(132.5 ± 7.5) × 10^4^/mL; P < 0.001] compared with the control and HCA groups. In the HCA-IR and IR-HCA groups, the infiltrated cells in aqueous humor significantly decreased [P < 0.01 and P < 0.001, respectively; (56.67 ± 16.14) and (38.75 ± 15.02) × 10^4^/mL, respectively], compared with those in the IR group. The protein concentration in the aqueous humor was significantly higher in the IR group (9.19 ± 1.64 mg/mL; P < 0.001) than in the control and HCA groups. Compared with the IR group, the IR-HCA group attenuated the protein concentration increase in the aqueous humor (5.94 ± 0.31 mg/mL; P < 0.05; Fig 1B). These findings suggest that HCA induction after I/R injury has anti-inflammatory effects in vivo.

### HCA protected against I/R-induced retinal thinning and dysfunction in vivo

**Fig 2:**
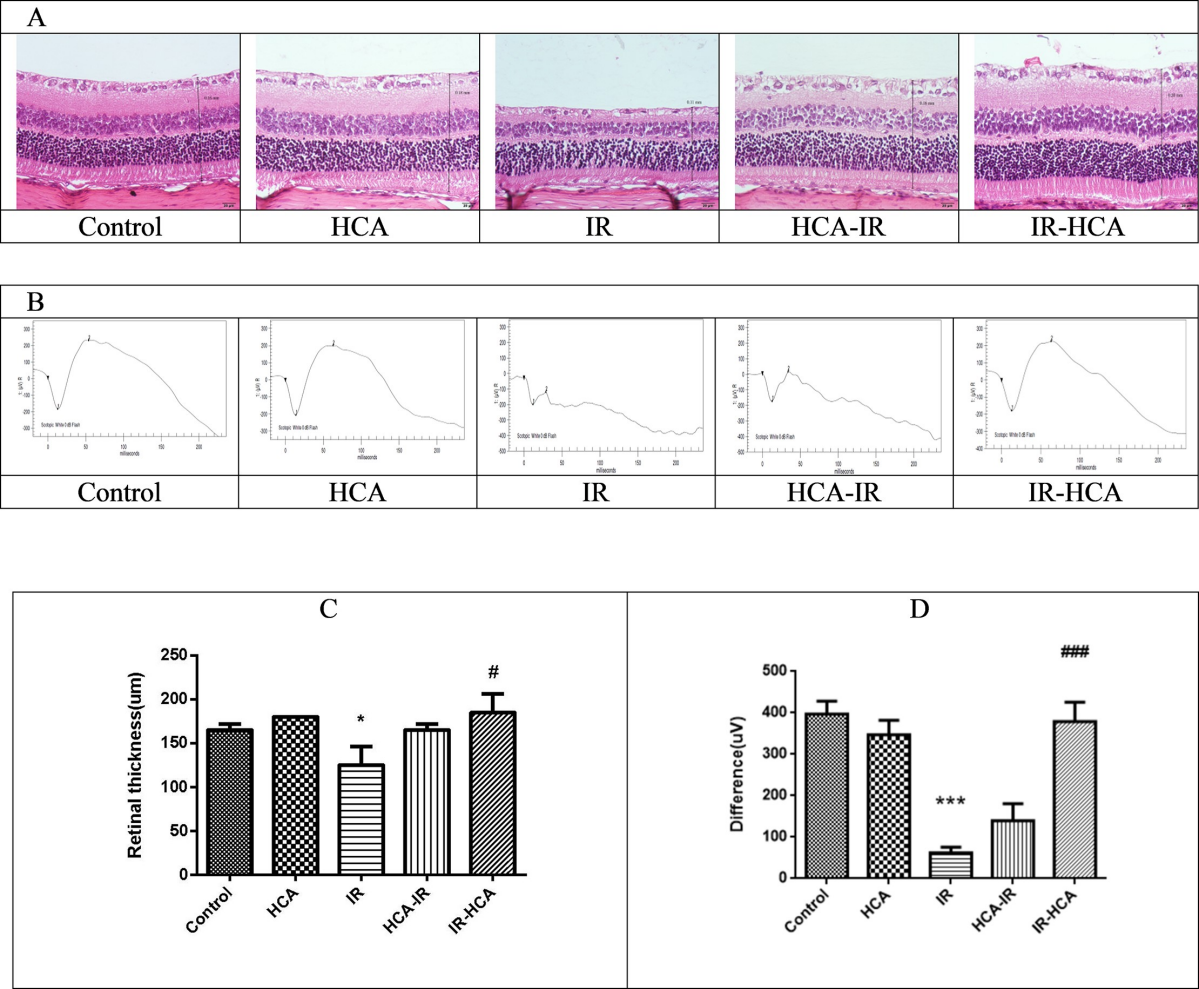
(A) Hematoxylin and eosin staining of the retinas. Representative photomicrographs showing the histologic appearance of retinas in the five groups. Significant decrease in the mean retinal thickness is visible in the IR group. (B) Representative ERG waveforms from the five groups. I/R injury significantly reduced the the α and β wave amplitudes. (C)Whole retinas were significantly thicker in the IR-HCA group than in the IR group. (D) ERG revealed significant recovery in the difference of cursor of α and β waves in the IR-HCA group. The results are mean ± SEM of three independent experiments. ***P < 0.001, *P < 0.05 versus control group; ###P < 0.001, #P < 0.05 versus IR group.

In the rats, retinal histopathological change and retinal function were examined after 7 days of I/R injury induction. Photomicrographs of the retina in the five groups are illustrated in Fig 2A. The retinal thickness in the IR group was significantly lower than that in the control and HCA groups. The mean retinal thickness in the IR-HCA group was significantly thicker than that in the IR group (Fig 2C).

The retinal function was evaluated by ERG in all groups (Fig 2B). In the control and HCA group, a typical dark-adapted scotopic ERG was observed. In the IR group, retinal ischemia contributed to a substantial decrease in α- and β-wave amplitudes. The difference in the cursor of α and β waves was significantly lower in the IR group (61.90 ± 5.66 μV) than in the control group (396.7 ± 12.98 μV; P < 0.001). A significant recovery in this difference was noted in the IR-HCA group (379.0±19.05 μV). HCA application reversed the amplitude reduction elicited by retinal ischemia 7 days of I/R injury induction, particularly in the IR-HCA group.

### HCA reduced retinal cell apoptosis

**Fig 3:**
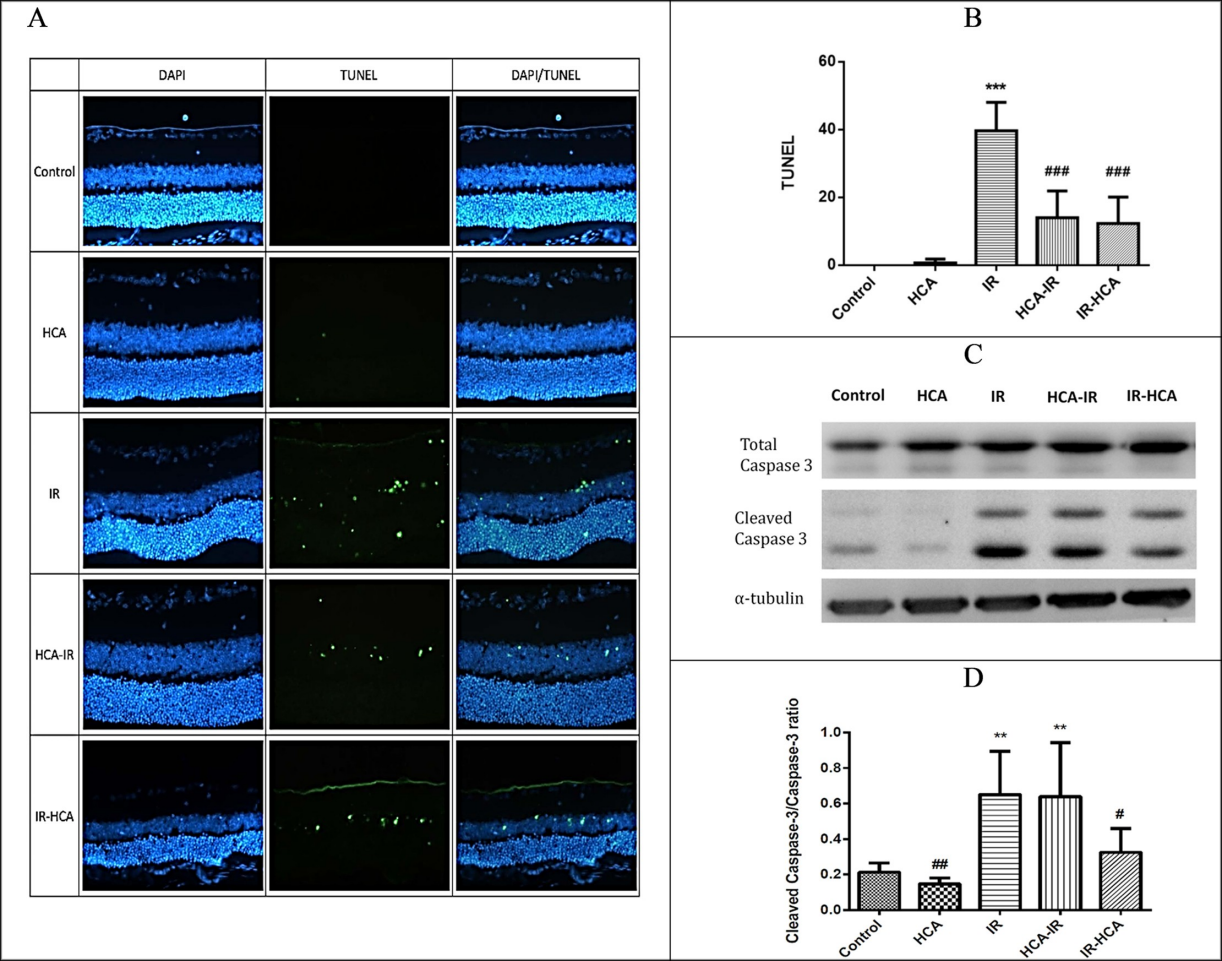
(A) Colocalization in TUNEL (apoptosis, green) and 4′,6-diamidino-2-phenylindole (DAPI; nuclei, blue).(B)Number of TUNEL-positive cells significantly decreased in both IR-HCA and HCA-IR groups. (C and D) Effects of HCA on the expression of the caspase-3 in the five groups. The results are mean ± SEM of three independent experiments. ***P < 0.001, **P < 0.01 versus control group; ###P < 0.001, ##P < 0.01, #P < 0.05 versus IR group.

Photomicrographs of TUNEL-positive cells in the retina of the rats in the five groups 24 hours after I/R injury are presented in Fig 3A. The number of TUNEL-positive cells was 39.67 ± 4.84/mm^2^, 14.00 ± 4.58/mm^2^, and 12.33 ± 4.49/mm^2^ in the IR, HCA-IR, and IR-HCA groups, respectively. The number of TUNEL-positive cells increased after I/R injury, but it was significantly lower in both IR-HCA and HCA-IR groups. Thus, retinal neural cell apoptosis triggered by I/R injury can be prevented through HCA induction.

The zymogen caspase-3 remains inactive until cleaved by an initiator caspase after apoptotic signaling events have occurred. In the current study, the ratio of cleaved caspase-3/total caspase-3 was considered an apoptosis marker [31]. This ratio was considerably higher in the IR and HCA-IR groups than in the control and IR-HCA groups (Fig 3C and 3D).

### HCA increased cell survival and inhibited oxidative stress-induced RGC-5 cell apoptosis

**Fig 4:**
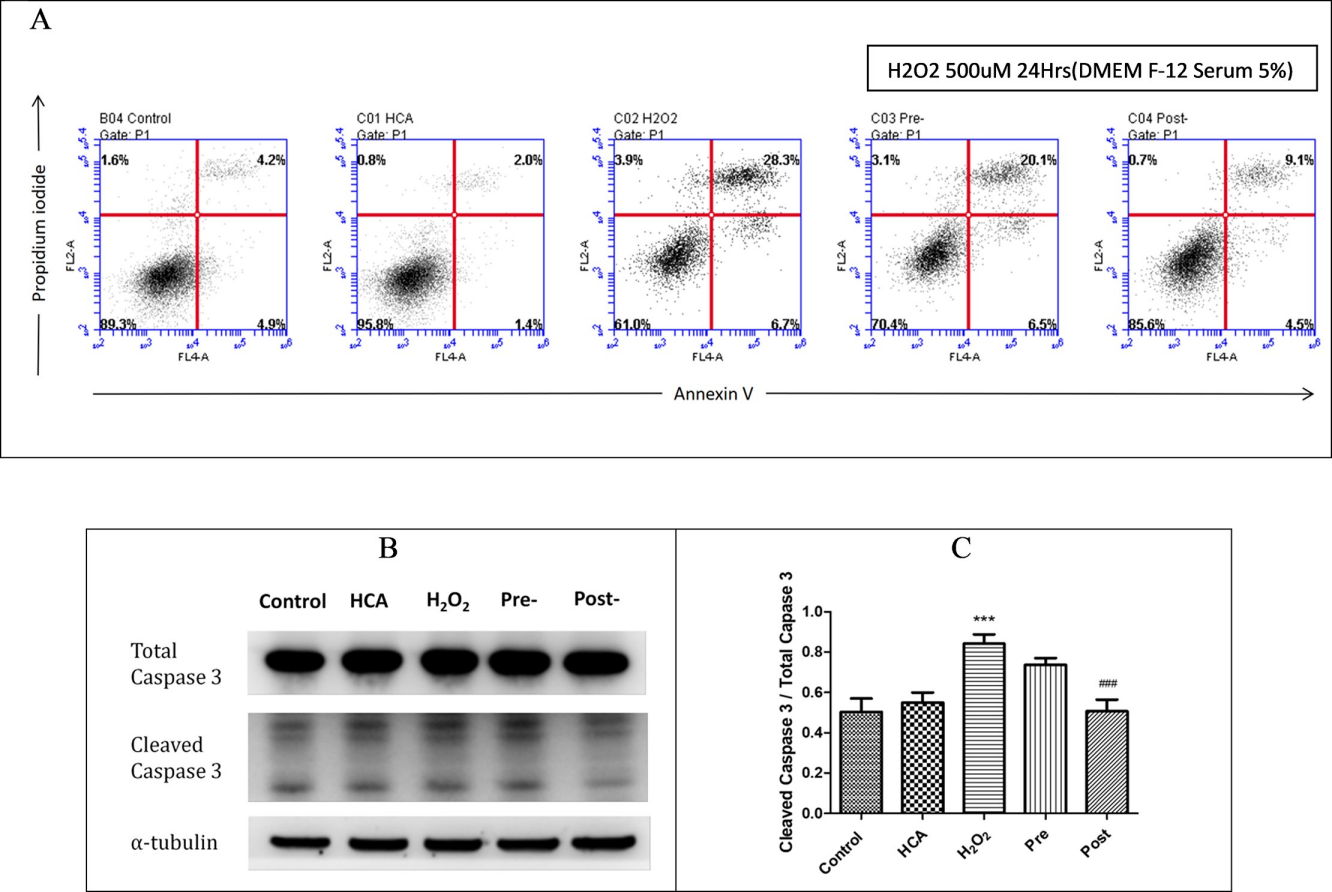
(A) Annexin V/PI flow cytometry of RGC-5cells in the five groups. The upper right quadrants (Annexin V+/PI+) represent apoptotic/necrotic cells, and the lower right quadrants (Annexin V+/PI−) represent early apoptotic cells.(B and C) Ratio of cleaved caspase-3/total caspase-3 was markedly increased by H_2_O_2_ compared with the control and post groups. The results are mean ± SEM of three independent experiments. ***P < 0.001 versus control group; ###P < 0.001 versus H_2_O_2_ group.

Reperfusion injury is associated with the formation of toxic ROS, including superoxide O_2_^−^, OH radicals, HOCl, H_2_O_2_, and nitric oxide-derived peroxynitrite [5]. An in vitro H_2_O_2_-induced apoptosis model was used to explore the effect of the oxidative stress of ROS induced by I/R injury. The RGC-5cells were treated with H_2_O_2_ (500 μM) for 60 minutes, and cell viability was measured through FACS flow cytometry (Fig 4A). As shown in Fig 4A, the apoptosis rates of the H_2_O_2_-treated and control groups were 35.0% and 9.1%, respectively. Before and after HCA induction, the apoptosis rates decreased to 26.6%and 13.6%, respectively, implying that HCA induction had a protective effect against ROS-induced apoptosis.

There was a significant increase of the ratio of cleaved caspase-3/caspase-3 in H_2_O_2_ groups compared with the control and post groups (Fig 4B and 4C).These findings revealed that HCA induction after H_2_O_2_ exposure had a protective effect against the H_2_O_2_-induced oxidative stress of RGC-5 cell apoptosis. In addition, studies have reported that apoptosis is induced by intracellular Ca^2+^ overloading through the opening of the mitochondrial permeability transition pore and ROS overproduction during reperfusion [32]. We provided evidence that HCA can reduce ROS generation and intracellular calcium release in vitro (S1 Fig). Taken together, these findings indicate that HCA induction after H_2_O_2_ exposure improved cell survival and reduced apoptosis by reducing ROS formation and ROS-associated calcium release in RGC-5 cells.

### HCA downregulated NF-κB and MAPK signals in vitro and in vivo

**Fig 5:**
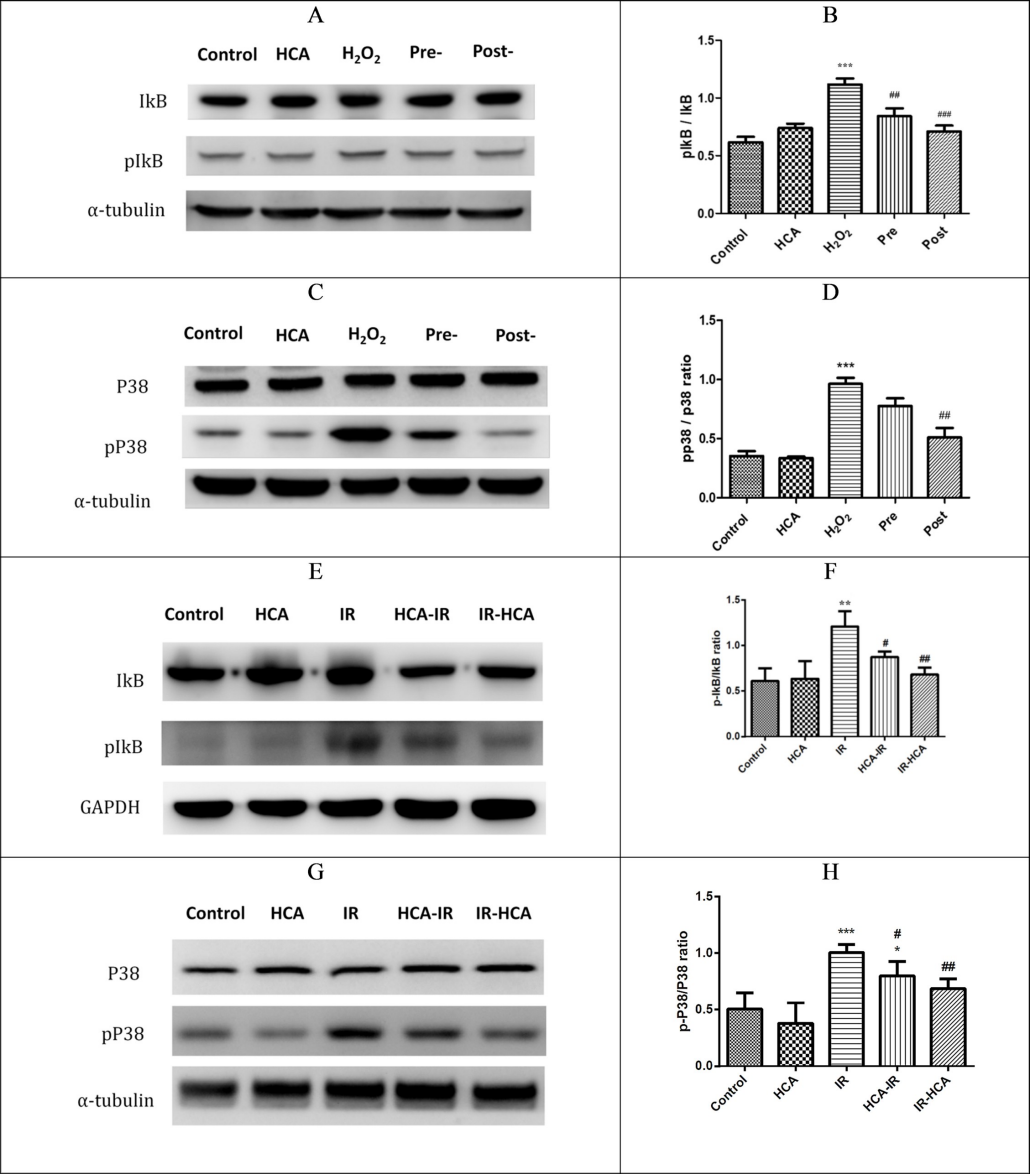
(A) Representative Western blots for the effects of HCA induction on the in vitro IκB expression in the five groups. (B) Effects of HCA induction on the IκB (pIκB/IκB ratio) in the five groups in vitro. (C and D) Activation of P38MAPK pathways in the five groups in vitro. The results are mean ± SEM of three independent experiments. ***P < 0.001 versus control group; ###P < 0.001, ## P<0.01 versus H_2_O_2_ group. (E) Representative Western blots for the effects of HCA induction on the expression of IκB in the five groups in vivo. (F) Effects of HCA on the IκB (pIκB/IκB ratio) in the five groups in vivo. (G and H) Activation of p38 MAPK in the five groups in vivo. The results are mean ± SEM of three independent experiments. ***P < 0.001,**P < 0.01,*P < 0.05 versus control group; ##P < 0.01, #P < 0.05 versus IR group.

I/R injury are also characterized by changes in the activity of intracellular signaling molecules. Several studies have shown that the anti-inflammatory effect of HCA is associated with the attenuation of NF-κB signaling [33, 34]. In our results, the ratios of phosphorylated IκB (pIκB) to IκB was used to represent the NF-κB signaling in ROS-induced RGC-5 damage or Sprague Dawley rats with I/R injury [35]. In vitro, the pIκB/IκB ratios significantly decreased in both pre and post groups compared with the H_2_O_2_ group (Fig 5A and 5B). In vivo, the pIκB/IκB ratio was significantly decreased in both HCA-IR and IR-HCA groups compared with the IR group (Fig 5E and 5F). Moreover, preconditioning with HCA was less effective than was applying HCA after injury in vivo and in vitro.

In various cell and organ systems, p38 MAPK activity increases on reperfusion [14, 36]. We further examined whether P38 MAPK signaling affects ROS damage in vitro and I/R injury in vivo. In vitro, treatment with HCA in the post groups significantly blocked the H_2_O_2_-induced phosphorylation of p38 MAPK (Fig 5C and 5D). In vivo, treatment with HCA after I/R injury significantly inhibited the phosphorylation of p38 MAPK (Fig 5G and 5H). Taken together, our findings indicated that HCA induction after I/R injury in vivo or after ROS damage in vitro can inhibit P38 MAPK signals and attenuate NF-κB activation.

### HCA enhanced the upregulation of HSP32 activity

**Fig 6:**
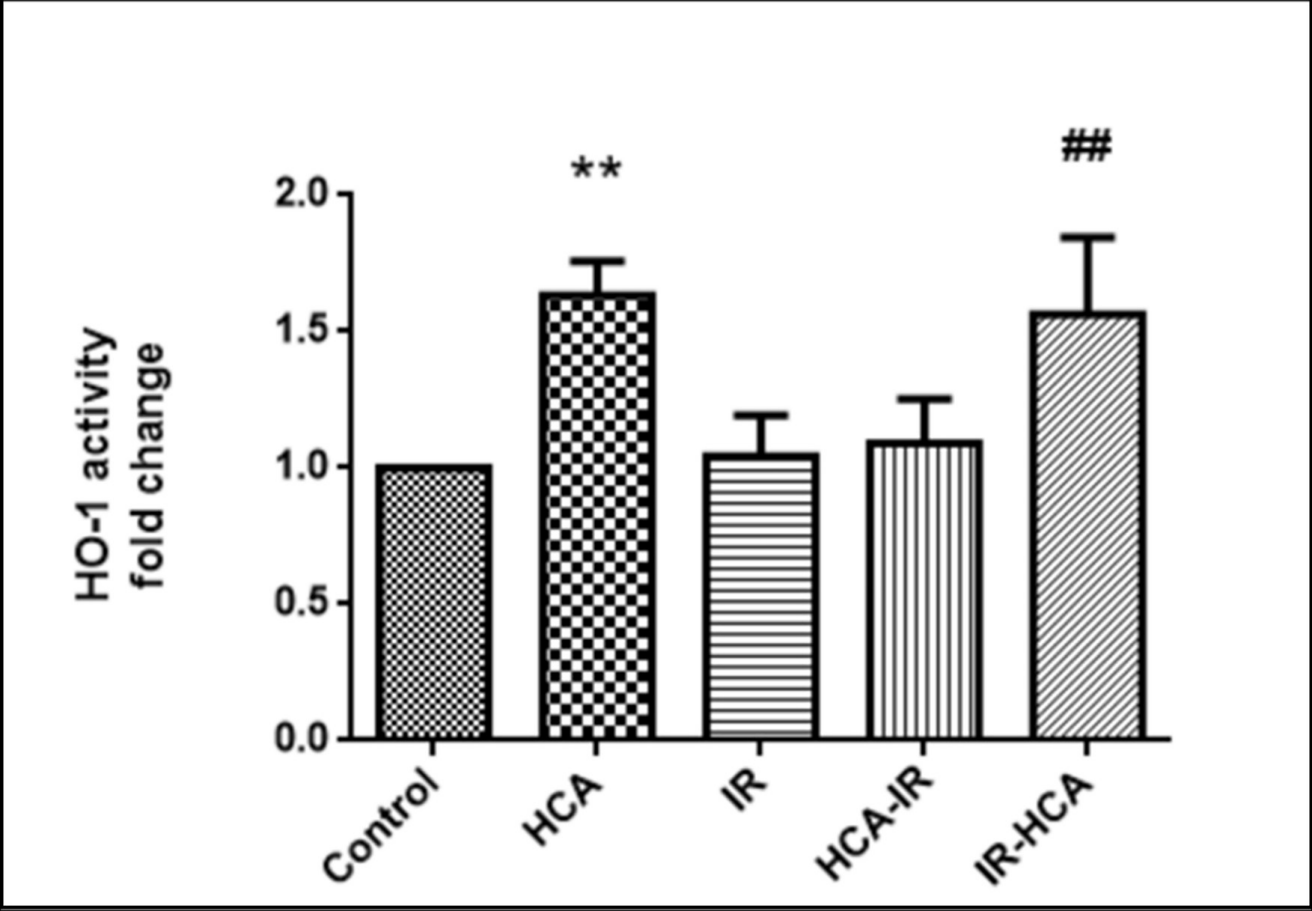
HSP32 expression was detected by measure protein activity. The results are mean ± SEM of three independent experiments. **P < 0.01 versus control group; ##P < 0.01 versus IR group.

When subjected to stress, a group of HSPs that play an important role in cell protection is induced by the affected cells [37]. These proteins provide protection against subsequent insults [38, 39]. Overexpression of HSP32, the inducible form of HO, has been shown to protect cells from I/R injury [22, 40]. We investigated whether their expression affects ROS damage in I/R injury. HSP32 expression was detected by measuring its activity; it was significantly increased in the HCA and IR-HCA groups (Fig 6).

## Discussion

To the best of our knowledge, the protective effects of HCA on retinal I/R injury and the underlying mechanisms have not been investigated. Various ocular diseases, including diabetic retinopathy, ophthalmic artery occlusion, central retinal artery occlusion, and acute angle-closure glaucoma, are associated with retinal I/R injury. Here, we investigated the in vivo effects of HCA induction in an artificially produced I/R-induced retinal damage model. The rat retinal I/R injury model mimics acute glaucoma and is a well-known animal model for studying retinal cell damage after ischemic insult [41]. We found that HCA induction provided protection against ROS production as well as retinal cell apoptosis and inflammation after I/R injury. Moreover, in the experimental model of I/R injury, HCA induction not only alleviated anterior chamber inflammation, thereby attenuating the reduction of retinal thickness, but also preserved retinal function to restore the ERG response to near-normal. In addition, our study indicated that HCA induction suppressed inflammation by the downregulation of NF-κB signaling in vitro and in vivo. This protective potential of HCA has been shown in various experimental models: Nardelli et al. indicated that HCA decreased lung and kidney cell apoptosis [42], and Masterson et al. observed that HCA reduced inflammation and lung injury in vivo and reduced NF-κB activation predominantly by inhibiting the activation and intrinsic activity of IκB kinase-β [33]. Our study is the first to demonstrate that HCA induction after I/R injury can provide retinal protection in a well-known animal model.

Substantial evidence has implicated ROS formation as a key pathogenic event in retinal I/R injury [5, 43]. To investigate the mechanism of protective effect of HCA induction, we subjected the retinal neural cell line RGC-5 to oxidative stress in vitro. In our study, HCA induction after H_2_O_2_ exposure could improve cell survival and reduce apoptosis by preventing intracellular calcium release and ROS overproduction in RGC-5 cells (S1 Fig). ROS act as second messengers in intracellular signaling cascades that control cell growth, proliferation, migration, and apoptosis [44]. Numerous cellular stimuli induce ROS production or activate MAPK pathways, but the exact underlying pathophysiological mechanisms remain unknown [45, 46]. H_2_O_2_ activates MAPK pathways via the activation of growth factor receptors in several cell types [47]. Consistent with this finding, in the present study, P38 MAPK pathways were activated in the H_2_O_2_-treated RGC-5 cells and rat retinas after I/R injury. Notably, HCA blocked the phosphorylation of p38 MAPK both in vivo and in vitro, implying that HCA can downregulate H_2_O_2_ or I/R injury-induced MAPK pathway activation.

In summary, we provide convincing evidence to suggest that HCA induction contributes to protection against retinal cell apoptosis and ocular inflammation; it also attenuated retinal thinning and preserved retinal function after I/R injury. Furthermore, HCA induction after H_2_O_2_ exposure showed consistent protection effects in RGC-5 cells. The protective effect of HCA may derive from its role in suppressing ROS overproduction and inhibiting NF-κB activation, which thus reduce proinflammatory cytokine expression [22, 33, 34]. In our study, HCA induction reduced the pIκB/IκB ratio both before and after injury in the I/R injury model and in RGC-5 cells. Preconditioning with HCA was less effective compared with postconditioning (Fig 5). Preconditioning modestly reduced the pIκB/IκB ratio but did not alter the effect of cell survival. Masterson et al. demonstrated that HCA directly reduces the kinase activity of the IKK complex and reduces IKK-mediated phosphoinactivation of IκBα [33]. Thus, the inhibition of the IKK–IκBα interaction is a key mechanism by which HCA suppresses canonical NF-κB activation in response to stimuli. Takeshita et al. demonstrated that isocapnic acidosis as well as buffered hypercapnia attenuated endotoxin-induced canonical NF-κB activation by suppressing IκBα degradation, although the degree of inhibition caused by isocapnic acidosis or buffered hypercapnia was smaller than that observed for HCA [34]. However, NF-κB signaling is complex; molecular mechanisms underlying the beneficial effects of HCA and the relative contribution of elevated CO_2_ levels or associated acidosis to this response remain poorly understood. We examined the effect of HCA on the retinal I/R injury in the presence and absence of NF-κB inhibition.

Retinal I/R injury comprises a self-reinforcing destructive cascade involving neuronal depolarization, calcium influx, and oxidative stress initiated by energy failure and increased glutamatergic stimulation [48]. During I/R, superoxide production rapidly and continually increases with the induction of global ischemia [49]. Regarding oxidative stress, disturbances in the antioxidant defense system have been demonstrated in I/R injury. Considerable evidence has implicated that eliminating ROS has a therapeutic benefit [22, 50–52]. In our previous study, we explored the protective effects of glucosamine (GlcN) in retinal I/R injury. GlcN contributes to protection from ROS overproduction through the modulation of protein O-linked N-acetylglucosamine (O-GlcNAc) glycosylation. GlcN could increase the O-GlcNAc level; increased cellular O-GlcNAc levels are cytoprotective. The posttranslational attachment of O-GlcNAc to serine and threonine residues of nuclear and cytoplasmic proteins is recognized as a key regulator of diverse cellular processes [53].

Li et al. indicated that HSP32 overexpression augments HCA protective effects in retinal I/R injury [40]. Here, our results demonstrated that HCA induction upregulates HSP32 activity to modulate superoxide production after I/R injury but does not increase the O-GlcNAc level. In contrast to large HSPs, HSP32 act through ATP-independent mechanisms. We noted that HCA induction, which .attenuated inflammatory responses by inhibiting ROS formation and reducing NF-κB signaling pathway activation, could enhance the upregulation of HSP32 activity which had an antioxidant effect. These results are consistent with those of a study that found that HCA induction upregulated HSP32 activity and ameliorated I/R injury in the lungs [22]. Although the interactions between HSP32 activity and retinal cell function are complicated and remain unclear, these pathways might be critical in cell function and survival.

In conclusion, HCA induction significantly reduced the damage caused by retinal I/R injury in the Sprague Dawley rat model and alleviated oxidative stress injury in the retinal neural cell line RGC-5. In these models, HCA induction had several protective effects: antiapoptotic, anti-inflammatory, and antioxidative. Our findings indicate HSP32 is vital in the retinal protection by HCA. HCA may be a potential agent for retinal protection from ischemic retinal diseases, such as diabetic retinopathy, glaucoma, and other vascular-related retinopathies.

## Supporting information

S1 Fig HCA reduced ROS generation and ROS-associated calcium release. (DOC)

## References

[1] Osborne NN, Casson RJ, Wood JP, Chidlow G, Graham M, Melena J. Retinal ischemia: mechanisms of damage and potential therapeutic strategies. Prog Retin Eye Res. 2004;23: 91–147. 10.1016/j.preteyeres.2003.12.001 14766318

[2] Fujita R, Ueda M, Fujiwara K, Ueda H. Prothymosin-alpha plays a defensive role in retinal ischemia through necrosis and apoptosis inhibition. Cell Death Differ. 2009;16: 349–58. 10.1038/cdd.2008.159 18989338

[3] Anderson DR, Davis EB. Sensitivities of ocular tissues to acute pressure-induced ischemia. Arch Ophthalmol. 1975;93: 267–274. 804301 10.1001/archopht.1975.01010020277006

[4] Nucci C, Tartaglione R, Rombola L, Morrone LA, Fazzi E, Bagetta G. Neurochemical evidence to implicate elevated glutamate in the mechanisms of high intraocular pressure (IOP)-induced retinal ganglion cell death in rat. Neurotoxicology. 2005;26: 935–41. 10.1016/j.neuro.2005.06.002 16126273

[5] Szabo ME, Droy-Lefaix MT, Doly M, Braquet P. Free radical-mediated effects in reperfusion injury: a histologic study with superoxide dismutase and EGB 761 in rat retina. Ophthalmic Res. 1991;23: 225–234. 10.1159/000267107 1945294

[6] Kennedy TP, Rao NV, Hopkins C, Pennington L, Tolley E, Hoidal JR. Role of reactive oxygen species in reperfusion injury of the rabbit lung. J Clin Invest. 1989;83: 1326–1335. 10.1172/JCI114019 2467923 PMC303825

[7] Hangai M, Yoshimura N, Yoshida M, Yabuuchi K, Honda Y. Interleukin-1 gene expression in transient retinal ischemia in the rat. Invest Ophthalmol Vis Sci. 1995;36: 571–578. 7890488

[8] Kerr NM, Johnson CS, Zhang J, Eady EK, Green CR, Danesh-Meyer HV. High pressure-induced retinal ischaemia reperfusion causes upregulation of gap junction protein connexin43 prior to retinal ganglion cell loss. Exp Neurol. 2012;234: 144–152. 10.1016/j.expneurol.2011.12.027 22226601

[9] Garcia-Valenzuela E, Shareef S, Walsh J, Sharma SC. Programmed cell death of retinal ganglion cells during experimental glaucoma. Exp Eye Res. 1995;61: 33–44. 7556468 10.1016/s0014-4835(95)80056-5

[10] Rabacchi SA, Bonfanti L, Liu XH, Maffei L. Apoptotic cell death induced by optic nerve lesion in the neonatal rat. J Neurosci. 1994;14: 5292–5301. 8083737 10.1523/JNEUROSCI.14-09-05292.1994 PMC6577062

[11] Huang C, Cen LP, Liu L. Adeno-associated virus-mediated expression of growth-associated protein-43 aggravates retinal ganglion cell death in experimental chronic glaucomatous injury. Mol Vis. 2013;19: 1422–1432. 23825922 PMC3695761

[12] Valko M, Leibfritz D, Moncol J, Cronin MT, Mazur M, Telser J. Free radicals and antioxidants in normal physiological functions and human disease. Int J Biochem Cell Biol. 2007;39: 44–84. 10.1016/j.biocel.2006.07.001 16978905

[13] Alvarado J, Murphy C, Polansky J, Juster R. Age-related changes in trabecular meshwork cellularity. Invest Ophthalmol Vis Sci. 1981;21: 714–727. 7298275

[14] Meldrum DR, Dinarello CA, Cleveland JC Jr. Hydrogen peroxide induces tumor necrosis factor alpha-mediated cardiac injury by a P38 mitogen-activated protein kinase-dependent mechanism. Surgery. 1998;124: 291–296. 9706151 10.1067/msy.1998.90570

[15] Moynagh PN. Toll-like receptor signalling pathways as key targets for mediating the anti-inflammatory and immunosuppressive effects of glucocorticoids. J Endocrinol. 2003;179: 139–144. 14596665 10.1677/joe.0.1790139

[16] Laffey JG, Kavanagh BP. Carbon dioxide and the critically ill-too little of a good thing? Lancet 1999;354: 1283–1286. 10.1016/S0140-6736(99)02388-0 . 10520649

[17] Laffey JG, O’Croinin D, McLoughlin P, Kavanagh BP. Permissive hypercapnia-role in protective lung ventilator strategies. Intensive Care Med 2004;30: 347–356. 10.1007/s00134-003-2051-1 . 14722644

[18] Chonghaile MN, Higgins BD, Costello J, Laffey JG. Hypercapnic acidosis attenuates lung injury induced by established bacterial pneumonia. Anesthesiology 2008;109: 837–848. 10.1097/ALN.0b013e3181895fb7 18946296

[19] Nomura F, Aoki M, Forbess JM, Mayer JE Jr. Effects of hypercarbic acidotic reperfusion on recovery of myocardial function after cardioplegic ischemia in neonatal lambs. Circulation 1994;90: II321–II327. 7955274

[20] Li AM, Quan Y, Guo YP, Li WZ, Cui XG. Effects of therapeutic hypercapnia on inflammation and apoptosis after hepatic ischemia-reperfusion injury in rats. Chin Med J. 2010;123: 2254–2258. 20819675

[21] Vannucci RC, Towfighi J, Heitjan DF, Brucklacher RM. Carbon dioxide protects the perinatal brain from hypoxic-ischemic damage: an experimental study in the immature rat. Pediatrics 1995;95: 868–874. 7761212

[22] Wu SY, Li MH, Ko FC, Wu GC, Huang KL, Chu SJ. Protective effect of hypercapnic acidosis in ischemia-reperfusion lung injury is attributable to upregulation of heme oxygenase-1. PLoS One. 2013;8(9): e74742 10.1371/journal.pone.0074742 24040332 PMC3769390

[23] Tong N, Zhang Z, Gong Y, Yin L, Wu X. Diosmin protects rat retina from ischemia/reperfusion injury. J Ocul Pharmacol Ther. 2012;28: 459–466. 10.1089/jop.2011.0218 22509733 PMC3459007

[24] Hackbarth H, Kuppers N, Bohnet W. Euthanasia of rats with carbon dioxide—animal welfare aspects. Lab Anim. 2000;34(1): 91–96. 10.1258/002367700780578055 10759372

[25] Chang YH, Horng CT, Chen YH, Chen PL, Chen CL, Liang CM, et al Inhibitory effects of glucosamine on endotoxin-induced uveitis in Lewis rats. Invest Ophthalmol Vis Sci. 2008;49(12): 5441–5449. 10.1167/iovs.08-1784 18719082

[26] Gavrieli Y, Sherman Y, Ben-Sasson SA. Identification of programmed cell death in situ via specific labeling of nuclear DNA fragmentation. J Cell Biol. 1992;119(3): 493–501. 1400587 10.1083/jcb.119.3.493 PMC2289665

[27] Van Bergen NJ, Wood JP, Chidlow G, Trounce IA, Casson RJ, Ju WK, et al Recharacterization of the RGC-5 retinal ganglion cell line. Invest Ophthalmol Vis Sci. 2009;50(9): 4267–4272. 10.1167/iovs.09-3484 19443730

[28] Sirieix D, Delayance S, Paris M, Massonnet-Castel S, Carpentier A, Baron JF. Tris-hydroxymethyl aminomethane and sodium bicarbonate to buffer metabolic acidosis in an isolated heart model. Am J Respir Crit Care Med. 1997;155(3): 957–963. 10.1164/ajrccm.155.3.9117032 . 9117032

[29] Maines MD, Trakshel GM, Kutty RK. Characterization of two constitutive forms of rat liver microsomal heme oxygenase. Only one molecular species of the enzyme is inducible. J Biol Chem. 1986;261: 411–419. 3079757

[30] Kanski JJ, Bowling B, Nischal KK, Pearson A. Clinical ophthalmology: a systematic approach. 7th ed Edinburgh; New York: Elsevier/Saunders 2011.

[31] Duan H, Zhang X, Chai J, Hu Q, Liu L, Ma L, et al Apoptosis and death receptor signaling in diaphragm of burnt rats. J Surg Res. 2016;203(1): 6–14. 10.1016/j.jss.2016.01.035 27338528

[32] Görlach A, Bertram K, Hudecova S, Krizanova O. Calcium and ROS: a mutual interplay. Redox Biol. 2015;6: 260–271. 10.1016/j.redox.2015.08.010 26296072 PMC4556774

[33] Masterson C, O’Toole D, Leo AM, McHale P, Horie S, Devaney J, et al Effects and mechanisms by which hypercapnic acidosis inhibits sepsis induced canonical NF-κB signaling in the lung. Crit Care Med. 2016;44(4): e207–17. 10.1097/CCM.0000000000001376 26584194

[34] Takeshita K, Suzuki Y, Nishio K, Takeuchi O, Toda K, Kudo H, et al Hypercapnic acidosis attenuates endotoxin-induced nuclear factor-κB activation. Am J Respir Cell Mol Biol. 2003;29: 124–132. 10.1165/rcmb.2002-0126OC 12600832

[35] Viatour P, Merville MP, Bours V, Chariot A. *Ph*os*phorylation* of NF-kappaB and IkappaB proteins: implications in cancer and inflammation. Trends Biochem Sci. 2005;30: 43–52. 10.1016/j.tibs.2004.11.009 15653325

[36] Sucher R, Gehwolf P, Kaier T, Hermann M, Maglione M, Oberhuber R, et al Intracellular signaling pathways control mitochondrial events associated with the development of ischemia/reperfusion-associated damage. Transpl Int. 2009;22: 922–930. 10.1111/j.1432-2277.2009.00883.x 19413579

[37] Morimoto RI, Tissieres A, Georgopoulos C. The biology of the heat shock proteins and molecular chaperones. New York: Cold Spring Harbor Laboratory Press; 1994.

[38] Parsell DA, Lindquist S. The function of heat shock proteins as molecular chaperones in stress tolerance: degradation and reactivation of damaged proteins. Annu Rev Genet. 1993;27: 437–496. 10.1146/annurev.ge.27.120193.002253 8122909

[39] Georgopoulos C, Welch WJ. Role of major heat shock proteins as molecular chaperones. Annu Rev Cell Biol. 1993;9: 601–634. 10.1146/annurev.cb.09.110193.003125 8280473

[40] Li L, Du G, Wang D, Zhou J, Jiang G, Jiang H. Overexpression of heme oxygenase‐1 in mesenchymal stem cells augments their protection on retinal cells in vitro and attenuates retinal ischemia/reperfusion injury in vivo against oxidative stress. Stem Cells Int. 2017;2017: 4985323 10.1155/2017/4985323 28255307 PMC5309411

[41] Hartsock MJ, Cho H, Wu L, Chen WJ, Gong J, Duh EJ. A mouse model of retinal ischemia‐reperfusion injury through elevation of intraocular pressure. J Vis Exp. 2016;113: e54065. 10.3791/54065 PMC5091361 27501124

[42] Nardelli LM, Rzezinski A, Silva JD, Maron-Gutierrez T, Ornellas DS, Henriques I, et al Effects of acute hypercapnia with and without acidosis on lung inflammation and apoptosis in experimental acute lung injury. Respir Physiol Neurobiol. 2015;205: 1–6. 10.1016/j.resp.2014.09.007 25246186

[43] Izzotti A, Bagnis A, Saccà SC. The role of oxidative stress in glaucoma. Mutat Res. 2006;612: 105–114. 10.1016/j.mrrev.2005.11.001 16413223

[44] Thannickal VJ, Fanburg BL. Reactive oxygen species in cell signaling. Am J Physiol Lung Cell Mol Physiol. 2000;279: L1005–L1028. 10.1152/ajplung.2000.279.6.L1005 11076791

[45] Torres M, Forman HJ. Redox signaling and the MAP kinase pathways. Biofactors. 2003;17: 287–296. 12897450 10.1002/biof.5520170128

[46] Son Y, Cheong YK, Kim NH, Chung HT, Kang DG, Pae HO. Mitogen-activated protein kinases and reactive oxygen species: how can ROS activate MAPK pathways? J Signal Transduct. 2011;2011: 792639 10.1155/2011/792639 21637379 PMC3100083

[47] Guyton KZ, Liu Y, Gorospe M, Xu Q, Holbrook NJ. Activation of mitogen-activated protein kinase by H2O2. Role in cell survival following oxidant injury. J Biol Chem. 1996;271: 4138–4142. 8626753 10.1074/jbc.271.8.4138

[48] Osborne NN, Casson RJ, Wood JP, Chidlow G, Graham M, Melena J. Retinal ischemia: mechanisms of damage and potential therapeutic strategies. Prog Retin Eye Res. 2004;23: 91–147. 10.1016/j.preteyeres.2003.12.001 14766318

[49] N¨ap¨ankangas JP, Liimatta EV, Joensuu P, Bergmann U, Ylitalo K, Hassinen IE. Superoxide production during ischemia-reperfusion in the perfused rat heart: a comparison of two methods of measurement. J Mol Cell Cardiol. 2012;53: 906–915. 10.1016/j.yjmcc.2012.09.011 23036824

[50] Pan H, He M, Liu R, Brecha NC, Yu ACH, Pu M. Sulforaphane protects rodent retinas against ischemia-reperfusion injury through the activation of the Nrf2/HO-1 antioxidant pathway. PLoS ONE. 2014;9(12): e114186 10.1371/journal.pone.0114186 25470382 PMC4254947

[51] Chen L, Qi Y, Yang X. Neuroprotective effects of crocin against oxidative stress induced by ischemia/reperfusion injury in rat retina. Ophthalmic Res. 2015;54: 157–168. 10.1159/000439026 26437379

[52] Chen YJ, Huang YS, Chen JT, Chen YH, Tai MC, Chen CL, et al Protective effects of glucosamine on oxidative-stress and ischemia/reperfusion-induced retinal injury. Invest Ophthalmol Vis Sci. 2015;56(3): 1506–1516. 10.1167/iovs.14-15726 25655796

[53] McLarty JL, Marsh SA, Chatham JC. Post-translational protein modification by O-linked N-acetyl-glucosamine: its role in mediating the adverse effects of diabetes on the heart. Life Sci. 2013;92: 621–627. 10.1016/j.lfs.2012.08.006 22985933 PMC3528804

