# Supplementary material for: Protective effects of hypercapnic acidosis on Ischemia–reperfusion-induced retinal injury
Source: PLoS One. 2019 Jan 25;14(1):e0211185. doi: 10.1371/journal.pone.0211185 (PMC6347245; doi:10.1371/journal.pone.0211185)
Supplement: S1 Fig — (DOC) [file pone.0211185.s001.doc]

Supporting Information

Oxidative stress play a crucial role in the pathogenesis of I/R has been supported by many lines of evidence. Here, we showed HCA induction after H_2_O_2_ exposure inhibited ROS formation and ROS-associated calcium release in RGC-5 cells.

| S1A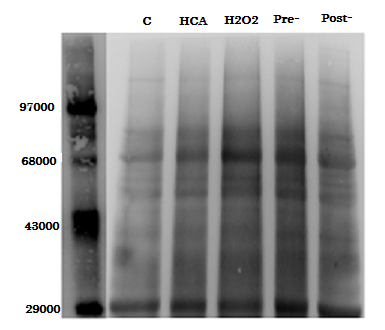 | S1C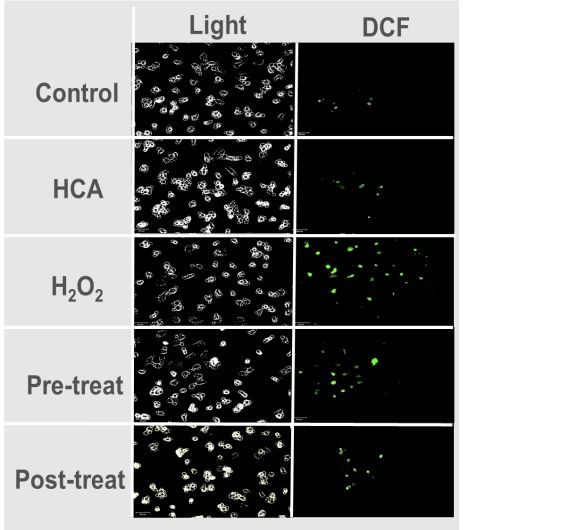 | S1E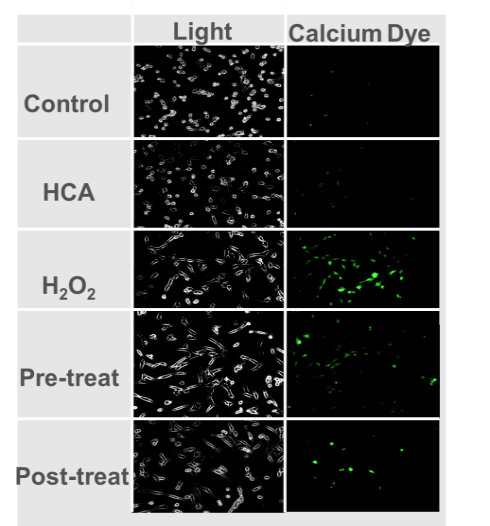 |
| --- | --- | --- |
| S1B  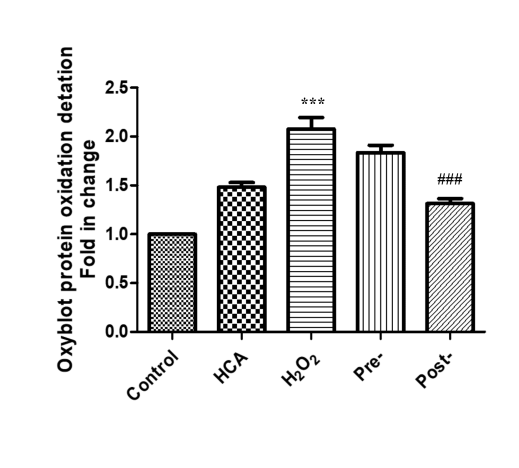 | S1D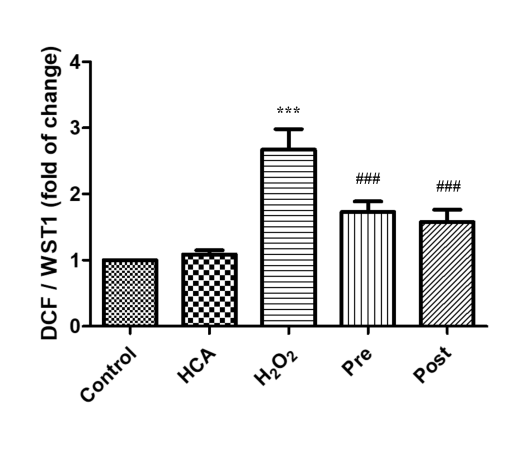 | S1F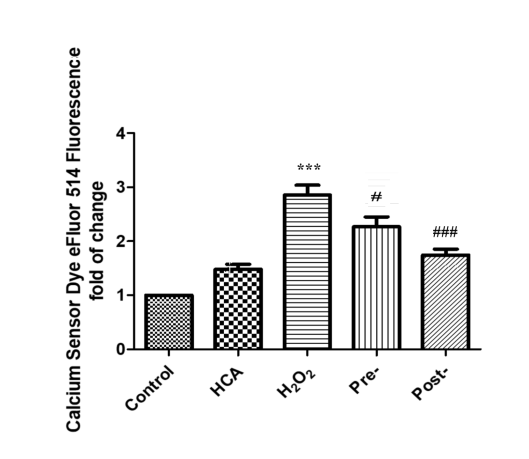 |

**Fig S1.** (A and B) Oxidation proteins were measured using Western blotting and quantified using the OxyBlot Protein Oxidation Detection Kit (Millipore). Protein detection was shown in fold change. (C and D) 2′,7′-dichlorofluorescin (DCF) was detected as an oxidation-sensitive fluorescent probe through fluorescence microscopy in the five groups. Cell viability was measured using a water-soluble tetrazolium salt (WST)-1 assay kit (Roche Diagnostics GmbH, Mannheim, Germany). (E and F) Calcium mobilization was assessed using Calcium Sensor Dye eFluor 514 in the five groups. The results are mean ± SEM of three independent experiments. ***P< 0.001 versus control group; ###P< 0.001, # P<0.05 versus H_2_O_2_ group.
